# Dinitrogen and Related Chemistry of the Lanthanides: A Review of the Reductive Capture of Dinitrogen, As Well As Mono- and Di-aza Containing Ligand Chemistry of Relevance to Known and Postulated Metal Mediated Dinitrogen Derivatives

**DOI:** 10.3390/ma3020841

**Published:** 2010-02-01

**Authors:** Michael G. Gardiner, Damien N. Stringer

**Affiliations:** School of Chemistry, University of Tasmania, Private Bag 75, Hobart TAS 7001, Australia; E-Mail: damien.stringer@utas.edu.au

**Keywords:** lanthanide, nitrogen reduction, nitrogen fixation, organolanthanide, imide, dinitrogen complexes, azobenzene reduction

## Abstract

This paper reviews the current array of complexes of relevance to achieving lanthanide mediated nitrogen fixation. A brief history of nitrogen fixation is described, including a limited discussion of successful transition metal facilitated nitrogen fixation systems. A detailed discussion of the numerous lanthanide-nitrogen species relevant to nitrogen fixation are discussed and are related to the Chatt cycle for nitrogen fixation.

## 1. Introduction to Nitrogen Fixation and Scope of Review

Man’s demand for chemically accessible nitrogen since the early 20^th^ century has far outstripped biology’s capacity to fix atmospheric nitrogen to ammonia. Necessarily, studies of synthetic chemical fixation were undertaken and resulted in the discovery by Fritz Haber of the first patented catalytic method to chemically fix nitrogen as ammonia, which was commercialised in 1910 by Carl Bosch [1,2,3]. The Haber-Bosch process, as it is now commonly known, fixes nitrogen *via* the use of high pressures of nitrogen and hydrogen over a heterogeneous Fe catalyst. The Haber-Bosch process has remained the primary synthetic means for the fixation of nitrogen to ammonia and now contributes half of the total required nitrogen input to world agriculture. Because of the high pressure and temperature demands of the process, 1% of the world’s total energy supply is consumed to satisfy society’s nitrogen demands [4].

**Figure 1:**
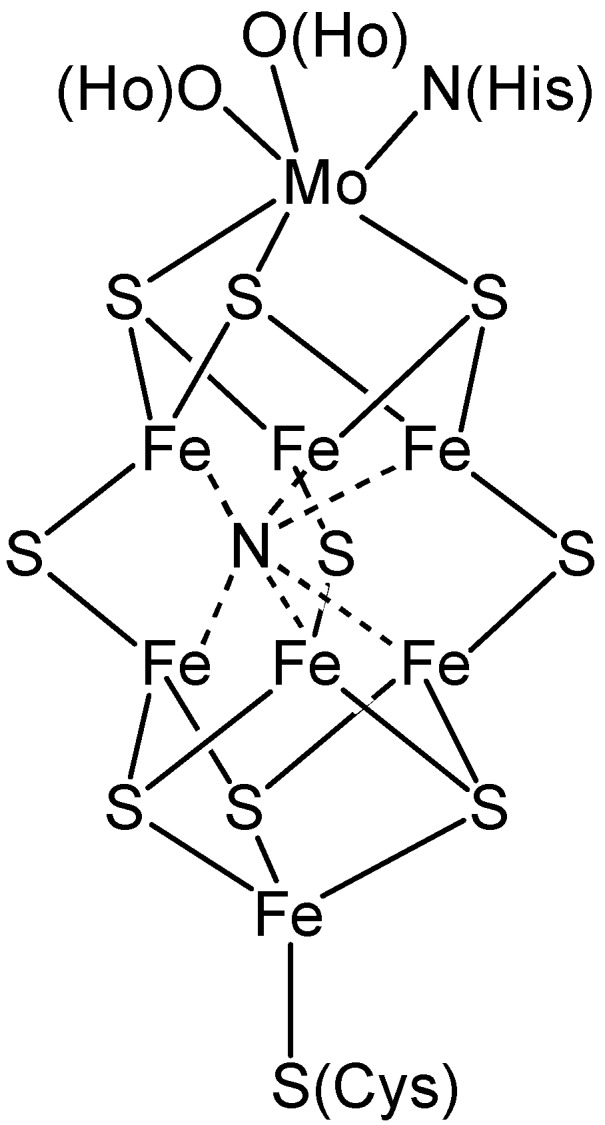
Model of the FeMo cofactor of nitrogenase adapted from Smith [4]. Ho = homocitrate, His = histamine, Cys = cysteine.

In this review we have focused on nitrogen capture chemistry based on organolanthanide systems. We have widened the scope of systems discussed beyond those species occurring in the Chatt cycle or isolated in extensive studies by Schrock, *vide infra*, given the bias of research activity in the field and the inherent reactivity of the lanthanides, which do not of course mirror that of the transition metals featuring in biological nitrogen fixation. A range of synthetic pathways have been presented, beyond direct reduction of nitrogen; with reduction and metallation routes from nitrogen containing organic and inorganic species being presented. Included are aryl/alkyl substituted analogues of complexes as postulated as occurring in the Chatt cycle, e.g., -N-N(H)R, but we have omitted discussion of chemistry where the substituent alters the bonding nature of the nitrogen- or dinitrogen-based ligand, such as the recently published hydrazonido, -NHN=CPh_2_, complexes [5]. We have not extended the discussion to include nitrogen reductions using lanthanide themselves (elemental form) or simple low valent species, but note that these species have been studied [6]. A personal account of part of this field was published in 2005 which described the lanthanide-based nitrogen reduction chemistry discovered in the research group of Evans until 2004 [7].

## 2. Biological Nitrogen Fixation

Biologically, nitrogen is fixed by a limited group of microbes at atmospheric pressures and temperatures by the nitrogenase enzyme. The contrast in energy requirements between the Haber-Bosch process and biological nitrogen fixation has resulted in significant research focus over the last 50 years to understand the nitrogen fixation mechanism within the nitrogenase enzyme [8]. Through studies of this nature, four different nitrogenase enzymes have been characterized [7,8,9,10,11], with varying metal co-factors present, containing Fe in each case and either Mo or V depending on the bio-availability of each metal to the microbe [9,12,13], Figure 1. The exploratory studies of nitrogenase enzymes have still not conclusively established the mechanism of nitrogen fixation, but have been influential in determining the metals that have been most extensively studied for chemical nitrogen fixation by synthetic methods.

## 3. Synthetic Transition Metal Mediated Nitrogen Fixation

**Scheme 1:**
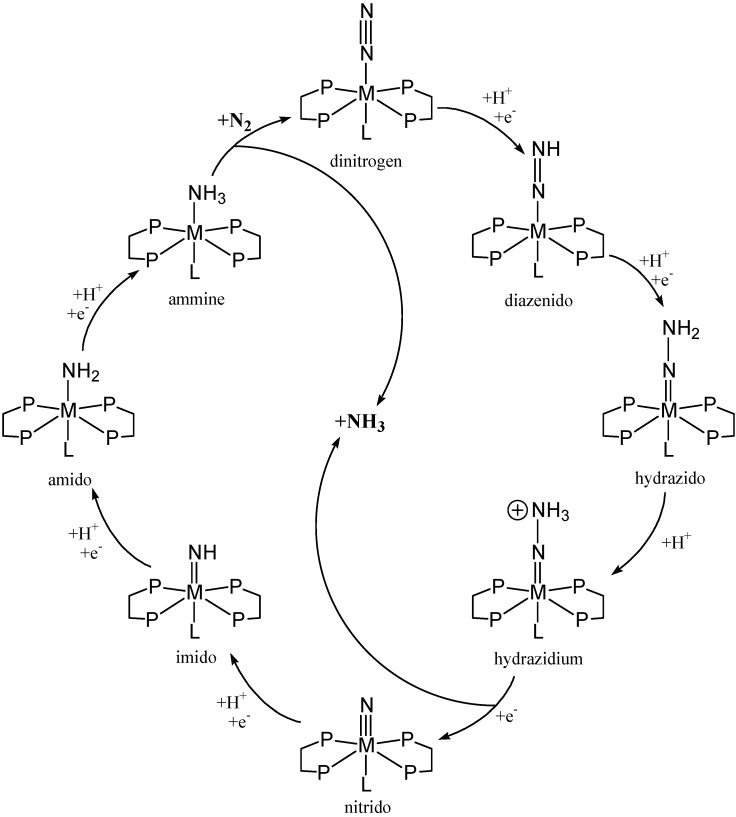
Chatt cycle for dinitrogen fixation at a single metal centre adapted from MacKay [23].

Allen and Senoff isolated the first dinitrogen complex of a transition metal, [Ru(NH_3_)_5_(N_2_)]^2+^ (**I**) [14], in 1965 from the aqueous reaction of hydrazine with ruthenium trichloride, and since this initial discovery, transition metal complexes displaying promise for achieving nitrogen fixation have been reported in abundance [15,16,17,18,19,20]. Early attempts of nitrogen fixation at a single metal centre focused on the use of Mo(0) and W(0) species. Through this early work, the Chatt cycle for nitrogen fixation was proposed as a hypothetical mechanism, Scheme 1. Several key intermediates have been identified (in some cases as *N*-substituted analogues) in this catalytic nitrogen fixation cycle, including M(N_2_), M–N=NH, M=N–NH_2_, M≡N, M=NH, M–NH_2_ and M(NH_3_) species. However, catalytic activity was not observed [21,22].

**Scheme 2:**
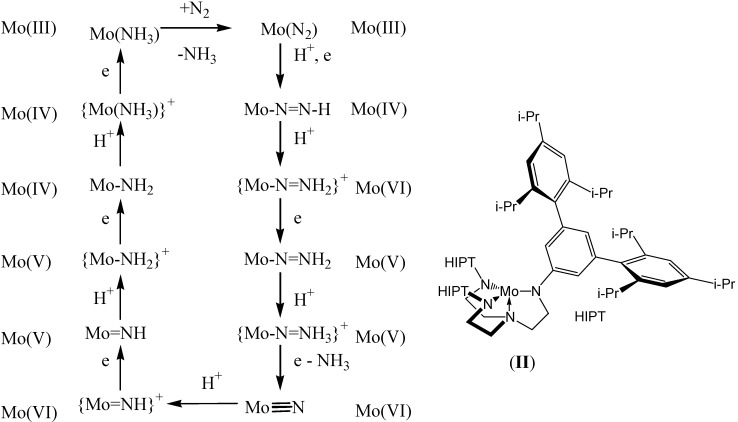
Schrock catalytic cycle for the Mo HIPT system adapted from Yandulov [25].

Following decades of research into transition metal dinitrogen chemistry, the first catalytic reductions of N_2_ to NH_3_ have recently been reported [24,25]. Of these, only that of Schrock has been rationalised *via* the characterisation of a number of intermediate species. The Mo HIPT system studied by Schrock, Scheme 2, was observed to involve several of the key intermediates proposed in the Chatt cycle from the 1960’s, and a catalytic cycle based on the characterised intermediate species has been proposed. The Mo centre within the cycle ranges in oxidation state from Mo^III^ to Mo^VI^.

The liberated NH_3_(*g*) in the Schrock cycle has been shown to inhibit catalysis as it competes with N_2_ for the vacant coordination site on [MoHIPT]. Furthermore, it is proposed that the activity of other transition metals such as W, Fe or V for N_2_ fixation with the HIPT system will be less than that observed for Mo [22].

**Scheme 3:**
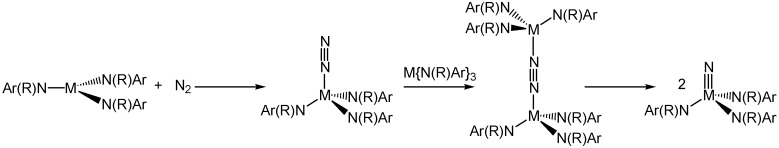
Mechanism for N_2_ cleavage by the MoN(R)Ar system [29,30].

Studies of bi-metallic systems with alternative mechanisms to the Chatt cycle have also lead to N_2_ cleaved species [26,27,28]. For instance, a molybdenum nitride complex was observed with the Mo(III) trisamide species, [Mo(NRAr)_3_], [R = C(CD_3_)_2_CH_3_, (**III**) Ar = 3,5-(Me)_2_C_6_H_3_, (**IV**)], whereby two [Mo(NRAr)_3_] molecules cooperatively reduced the N_2_ moiety without the need for the first three intermediate species in the Chatt cycle, Scheme 3. The reduction of N_2_ was observed to be stoichiometric, with catalytic activity not observed. Theoretical studies of this system have yielded detailed mechanistic knowledge [29,30].

Recent studies of Group 4 metal compounds have yielded a number of activated nitrogen species which have exhibited reactivity with carbon containing molecules, as well as hydrogen, though a catalytic system has yet to be found [31,32].

## 4. Lanthanide Mediated Dinitrogen Reduction

**Figure 2:**
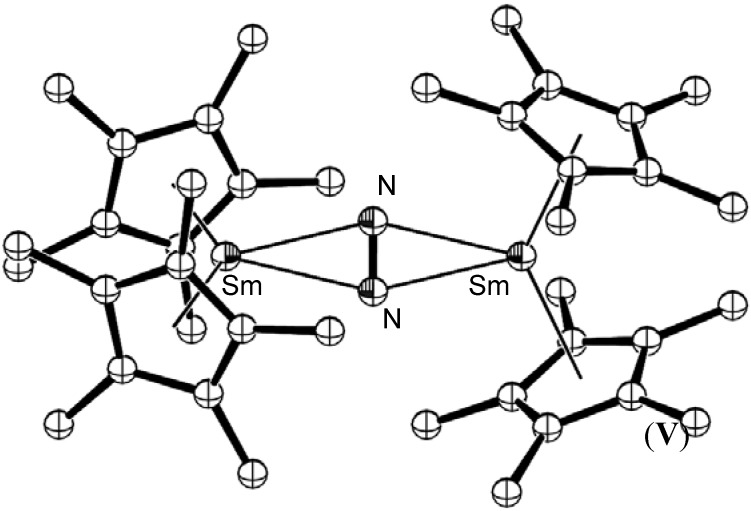
Molecular structure of side-on bound dinitrogen decamethylsamarocene complex **V** [34]. Figure generated from CCDC obtained coordinates. Atoms of arbitrary size. H atoms omitted for clarity.

Dinitrogen complexes of the transition metals have historically exhibited end-on bonding to one or more metal centres. Typically, weak activation of the N≡N bond is observed for end-on bound nitrogen molecules with single metal centres, such as the Schrock Mo(HIPT) system [33]. In 1998, Evans reported the synthesis of a novel side-on bound dinitrogen complex of decamethylsamarocene, [{(C_5_Me_5_)_2_Sm}_2_(µ_2_-N_2_)], (**V**), as shown in Figure 2 [34]. Whilst little activation of the dinitrogen bond was observed in this case (based on the N-N bond length), the possibility that side-on bound dinitrogen complexes could be synthesised led to renewed efforts utilising other metals to replicate the side-on bonding mode. This indeed led to the isolation of side-on bound dinitrogen complexes of other lanthanides, actinides, and the transition metals, Zr, Nb, Ta, Hf and Os [35]. From these studies, it has been shown that the side-on bonding mode can result in quite variable dinitrogen activation, ranging from weakly (N≡N bonds) to strongly (N=N and N-N bonds) activated species, as shown in Table 1.

**Table 1:** N_2_ activation according to bonding mode, adapted from Fryzuk [33,36].

N_2_ Binding Mode	Weak Activation	Strong Activation
End-on mononuclear	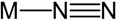	
End-on dinuclear	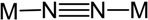	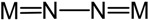
Side-on dinuclear	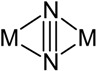	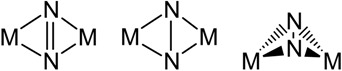
Side-on/End-on dinuclear		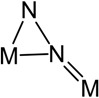

The novel planar side-on N_2_ binding mode exhibited by [{(C_5_Me_5_)_2_Sm}_2_(µ_2_-N_2_)], (**V**), coupled with the reducing power of the Sm^II^/Sm^III^ redox couple suggested that further studies utilising lanthanide based reducing agents may reveal insights into dinitrogen reduction chemistry [35].

**Figure 3:**
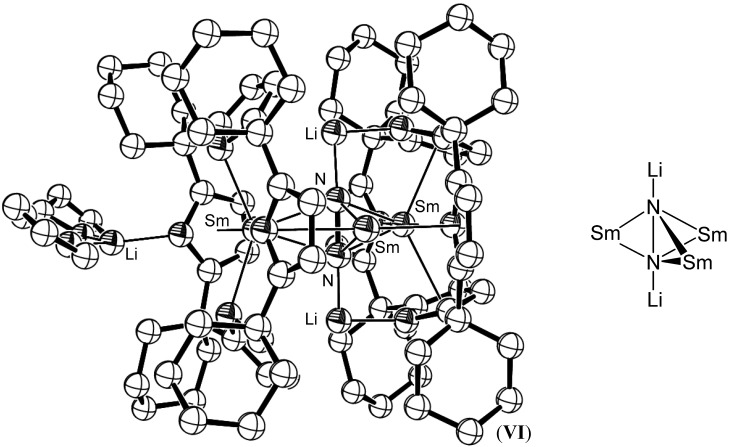
Strongly reduced (N_2_)^4-^ species in [{(*c*-hex_4_N_4_)_2_Sm_3_Li_2_}(µ^3^-N_2_) {Li(THF)_2_}^.^THF], (**VI**) [40]. Figure generated from CCDC obtained coordinates. Atoms of arbitrary size. H atoms are omitted for clarity. Also shown is a partial schematic of the binding observed for (N_2_)^4-^ fragment.

Subsequent studies have resulted in the formation of several Sm mediated reduced dinitrogen species and led to the extension of dinitrogen reduction chemistry to the majority of the remaining lanthanides [37]. In each case, the Ln(II) centre contributes one electron to the reduction of the dinitrogen bond, most often resulting in a bimetallic complex bridged by a dianionic reduced dinitrogen ligand, as shown for [{(C_5_Me_5_)_2_Sm}_2_(µ_2_-N_2_)], (**V**) [38]. For the strongly reducing lanthanide, dysprosium(II), a three-electron reduced dinitrogen species has also recently been reported [39]. Dinitrogen reduction was shown to be reversible in many cases, as for [{(C_5_Me_5_)_2_Sm}_2_(µ_2_-N_2_)], (**V**). Gambarotta reported the cooperative reduction of dinitrogen by four Sm(II) centres bound to macrocyclic porphyrinogen ligands which resulted in a tetraanionic reduced (N_2_)^4-^ species [{(*c*-hex_4_N_4_)_2_Sm_3_Li_2_}(µ^3^-N_2_){Li(THF)_2_}·THF], (**VI**), shown in Figure 3 (*c*-hex_4_N_4_ = *meso*-(CH_2_)_5_-calix-4-pyrollide), whereby the fourth samarium atom involved in the reduction formed the samarium(III) macrocyclic by-product, [(*c*-hex_4_N_4_)Sm(Cl){(Li(THF))_3_(µ^3^-Cl)}], (**VII**) [40]. Such strongly reduced species do not occur frequently from Ln(II) based reductions, as a number of metal centres are required to cooperatively reduce the dinitrogen substrate. Catalytic dinitrogen reduction systems utilising lanthanide metals are more commonly postulated to involve an external electron source [41].

**Figure 4:**
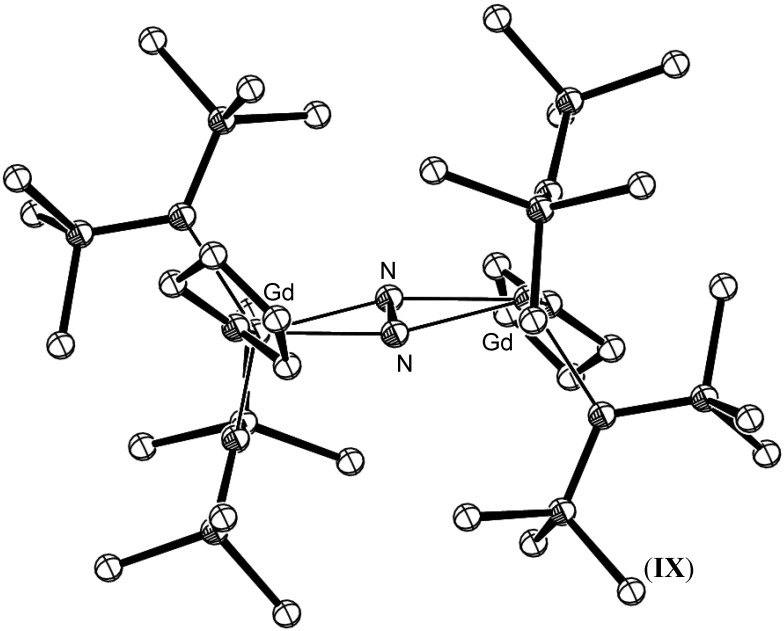
Reduced dinitrogen Gd complex, [{(THF)Gd(N(SiMe_3_)_2_)_2_}_2_N_2_], (**IX**) [44]. Disorder in one TMS group is not shown. Figure generated from CCDC obtained coordinates. Atoms of arbitrary size. H atoms omitted for clarity.

Gambarotta has also reported the synthesis of a four electron reduced N_2_ samarium porphyrinogen complex, [{(THF)_2_Li(Et_8_N_4_)Sm}_2_(N_2_)Li_4_)], (**VIII**), (Et_8_N_4_ = *meso*-octaethylcalix-4-pyrollide), which utilised Li as an external electron source for the reduction, with reduction of the nitrogen fragment also involving Sm(II)/Sm(III) oxidation [41]. Similarly, Floriani has reported the cooperative reduction of dinitrogen by *meso*-octaethyl porphyrinogen complexes of Pr and Nd in the presence of sodium [42]. Hetero-/multinuclear Lanthanide complexes such as these macrocyclic examples suggest that bimetallic complex formation and additional external electron sources greatly widen the range of reduced dinitrogen species that can be obtained for metals that do not have widely accessible oxidation states. Lanthanide mediated dinitrogen reduction utilising an external electron source has since been reported for the entire lanthanide series excepting Eu and Yb [43,44,45,46,47]. The lanthanide centre in each case was supported by bis(trimethylsilyl)amide ligands as shown for the Gd example shown in Figure 4.

The reactivity of Ln(III)/M mixtures with dinitrogen, coupled with Ln(II) dinitrogen reduction chemistry has shown the readiness with which the initial N_2_ reduction step can be undertaken by organolanthanide species. Studies of other N_2_ reduced species of the lanthanide elements have been limited, however a number of the key intermediates proposed in the Chatt cycle have been observed.

## 5. Reductions of RN=NR Species

**Figure 5:**
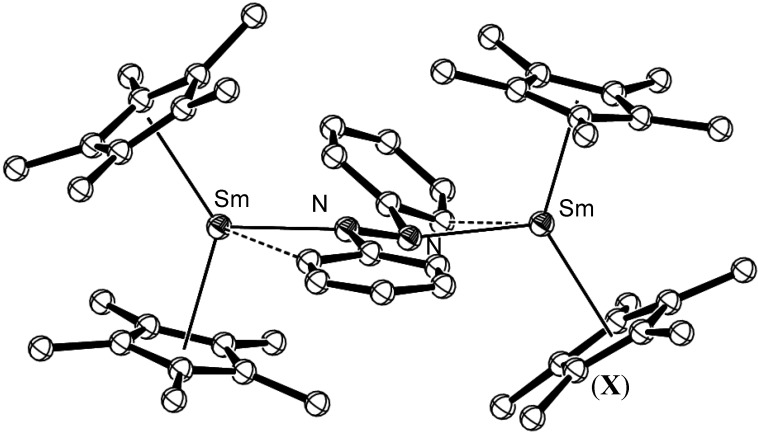
Molecular structure of [{(C_5_Me_5_)Sm}_2_(μ-η^1^:η^1^-N_2_Ph_2_)], (**X**). Figure generated from CCDC obtained coordinates. Atoms of arbitrary size. H atoms are omitted for clarity [48].

Organolanthanide complexes containing a dinitrogen moiety are limited primarily to those of anionic (N_2_)^n-^, n = 2, 3, 4, species from dinitrogen reduction, as discussed above [34,35,36,37,38,39,40,41,42,43,44,45,46,47], and reduced azobenzene compounds, (RNNR)^n-^, n = 1, 2, which relate to the diazenido and hydrazido species proposed in the Chatt cycle. The first organolanthanide azo complex, [{(C_5_Me_5_)Sm}_2_(μ-η^1^:η^1^-N_2_Ph_2_)], (**X**), contained two samarium(III) centres, bridged by a dianionic *trans*-azobenzene moiety, which exhibited lengthening in the N-C(phenyl) bonds of the azobenzene unit without lengthening (and inferred reduction) of the N=N bond (1.25(1) Å). Agostic hydrogen interactions to the *ortho* phenyl hydrogen atoms were observed [48], Figure 5.

**Figure 6:**
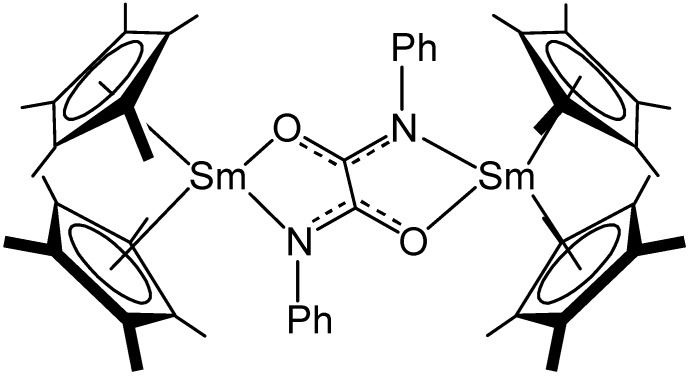
Representation of the structure of [{C_5_Me_5_)_2_Sm}_2_{(PhN)CO}_2_], (**XII**) [42].

Both [{(C_5_Me_5_)Sm}_2_(μ-η^1^:η^1^-N_2_Ph_2_)], (**X**) and its 3-tolyl analogue, [{(C_5_Me_5_)Sm}_2_(3-Me-H_4_C_6_NNC_6_H_4–_3-Me)], (**XI**), were found to exhibit reactivity with CO to form the corresponding *N,N*′-diarylphenoxamide ligands, which bridge each samarium centre through chelating N and O interactions to give [{C_5_Me_5_)_2_Sm}_2_{(PhN)CO}_2_], (**XII**), shown in Figure 6 and [{C_5_Me_5_)_2_Sm}_2_{[(3-MeC_6_H_4_N) (CO)}_2_], (**XIII**), respectively [48]. Reactivity patterns such as those observed for **X** and **XI** with CO highlight the readiness with which nitrogen centres from dinitrogen precursors may be derivatised and provides some insight into possible lanthanide mediated nitrogen reduction pathways.

**Scheme 4:**
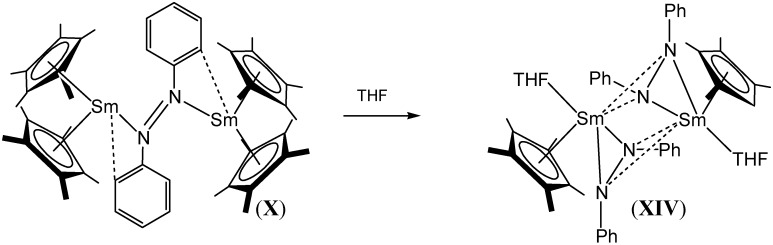
THF mediated transformation of [{(C_5_Me_5_)Sm}_2_(μ-η^1^:η^1^-N_2_Ph_2_)], (**X**) to [{(C_5_Me_5_)Sm(THF)}_2_{µ-η^2^: η^2^-N_2_Ph_2_}_2_], (**XIV**) [49].

[{(C_5_Me_5_)Sm}_2_(μ-η^1^:η^1^-N_2_Ph_2_)], (**X**), was also found to react in THF to give [{(C_5_Me_5_)Sm(THF)}_2_ {µ-η^2^:η^2^-N_2_Ph_2_}_2_], (**XIV**), as shown in Scheme 4, which exhibited a much longer N=N bond length of 1.44(1) Å [49].

**Figure 7:**
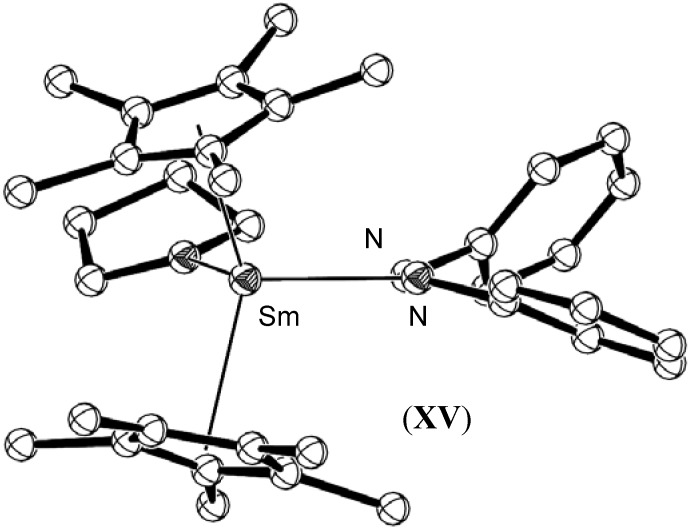
Molecular structure of [(C_5_Me_5_)_2_Sm(η^2^-N_2_Ph_2_)(THF)], (**XV**). Figure generated from CCDC obtained coordinates. Atoms of arbitrary size. H atoms and one of the two independent molecules in the asymmetric unit are omitted for clarity [49].

Further studies on this azobenzene system allowed the isolation of the singly reduced 1:1 *cis*-azobenzene complex [(C_5_Me_5_)_2_Sm(η^2^-N_2_Ph_2_)(THF)] (**XV**), shown in Figure 7. Efforts to synthesise the corresponding Yb analogue resulted in the isolation of the di-metallic ytterbium(III) species, [(C_5_H_5_)(THF)Yb]_2_[µ-η^2^:η^2^-N_2_Ph_2_]_2_ (**XVI**), which co-crystallised as a mixture with [(C_5_H_5_)_3_Yb(THF)] (**XVII**). The monometallic samarium complex **XV** showed no reactivity with CO, but in contrast to the bimetallic case of **X**, exhibited a lengthening of the N=N bond from 1.25 to 1.36 Å, without any change to the length of the N-C(phenyl) bonds [49].

Since the initial azobenzene reduction studies of Evans, a number of azobenzene lanthanide complexes have been reported. Bi-metallic systems are most common, with the azobenzene ligand bridging two lanthanide centres. However, a limited number of monometallic species have also been reported. 

**Figure 8:**
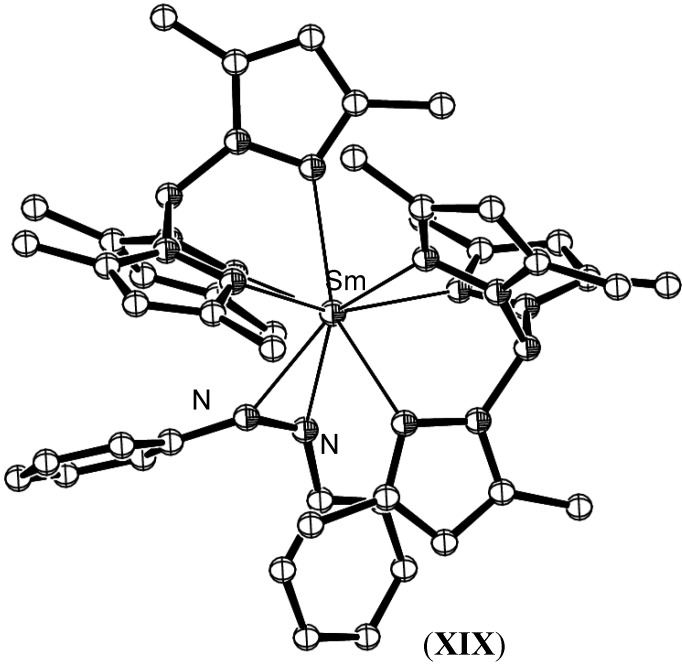
Molecular structure of [Sm{HB-(3,5-Me_2_pz)_3_}_2_(PhNNPh)], (**XIX**) [50]. Figure generated from CCDC obtained coordinates. Atoms of arbitrary size. H atoms are omitted for clarity.

Five monometallic lanthanide η^2^- azobenzene complexes have been reported, aside from those of Evans described above. The reaction of bis[hydrotris(3,5-dimethylpyrazolyl)borato]samarium(II), (**XVIII**), with azobenzene gave the monometallic singly reduced complex, [Sm{HB-(3,5-Me_2_pz)_3_}_2_(PhNNPh)], (**XIX**), as shown in Figure 8 [50]. The steric bulk of the hydrotris(3,5-dimethylpyrazolyl)borato ligand prevented further reduction by a second equivalent of the Sm(II) precursor complex, in contrast to the observed reactivity of the samarium C_5_Me_5_ system. The N=N bond exhibited similar lengthening to that observed for the decamethylsamarocene system, resulting in a bond length of 1.332(12) Å.

The divalent samarium aryloxide complex [Sm(OAr)_2_(THF)_3_], (**XX**) (Ar = C_6_H_2_-*tert*-Bu_3_-2,4,6) also formed a monometallic azobenzene complex, [Sm(OAr)_2_(PhNNPh)], (**XXI**), in which similar N=N bond lengthening to 1.358(11) Å was observed [51]. In this case, steric bulk did not appear to limit the reduction, as evident by the coordination of two THF molecules, as shown in Figure 12.

**Figure 9:**
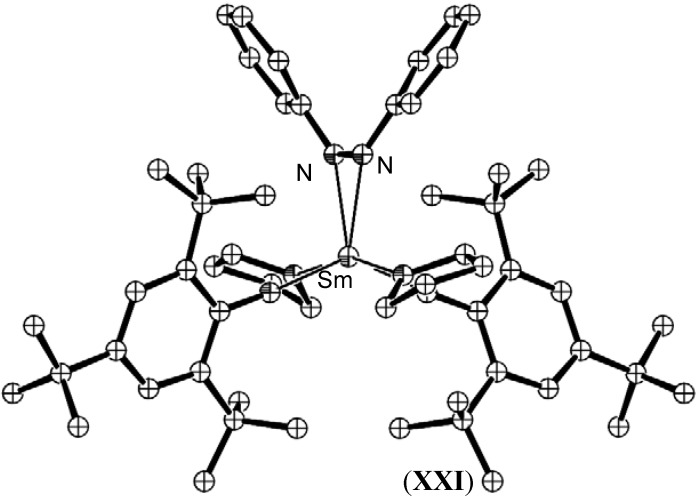
Molecular structure of [Sm(OAr)_2_(PhNNPh)], (**XXI**) [51]. Figure generated from CCDC obtained coordinates. Atoms of arbitrary size. H atoms and one diethyl ether molecule of crystallisation are omitted for clarity.

**Figure 10:**
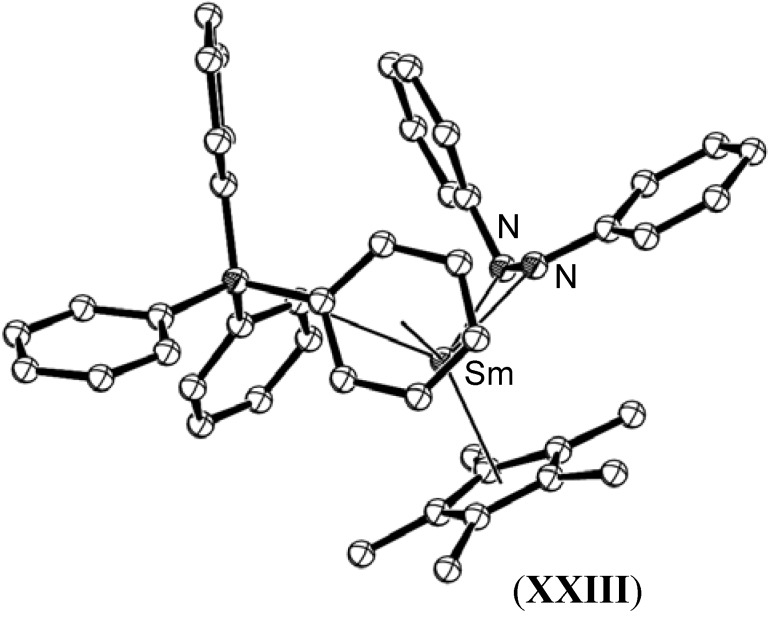
Molecular structure of [(C_5_Me_5_)Sm{(η^6^:η^1^-Ph)_2_BPh_2_}(PhNNPh)], (**XXIII**) [52]. Figure generated from CCDC obtained coordinates. Atoms of arbitrary size. H atoms are omitted for clarity.

Reaction of the divalent complex [(C_5_Me_5_)Sm(µ-η^6^:η^1^-Ph)_2_BPh_2_], (**XXII**), with azobenzene gave [(C_5_Me_5_)Sm{(η^6^:η^1^-Ph)_2_BPh_2_}(PhNNPh)], (**XXIII**), which exhibited a strongly reduced N=N bond with a length of 1.435(5) Å [52], which approaches the length of the N−N bond in hydrazine, as a consequence of the weaker electron donation from the (BPh_4_)^-^ moiety to the samarium(III) centre than in the analogous bis(pentamethylcyclopentadienyl) complex, [(C_5_Me_5_)_2_Sm(η^2^-PhNNPh)(THF)], (**XV**), described earlier, Figure 10.

**Figure 11:**
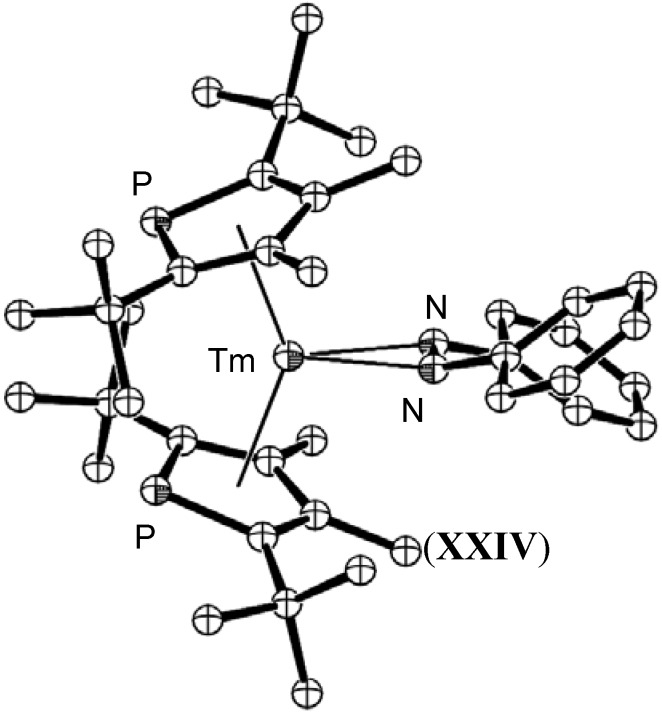
Molecular structure of [(PC_4_-2,5-*t*-Bu-3,4-Me)_2_Tm(PhNNPh)], (**XXIV**) [53]. Figure generated from CCDC obtained coordinates. Atoms of arbitrary size. H atoms are omitted for clarity.

Thulium and samarium(II) complexes of 2,5-di-*tert*-butyl-3,4-dimethylphospholide also gave reduced azobenzene complexes as shown in Figure 11 for [(PC_4_-2,5-*t*-Bu-3,4-Me)_2_Tm(PhNNPh)], (**XXIV**). Typical reduced bond lengths of 1.35 Å were observed in both complexes [53].

**Figure 12:**
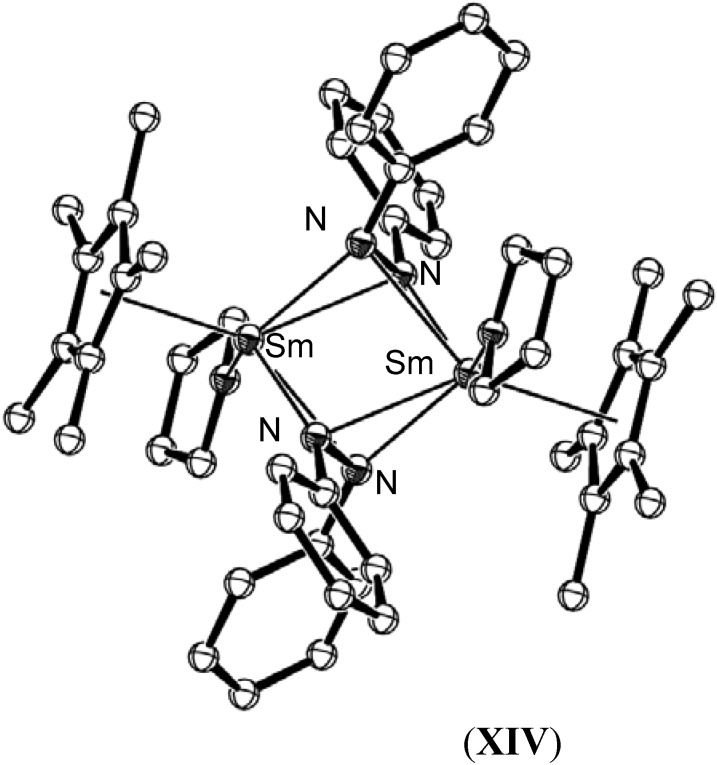
Molecular structure of [{(C_5_Me_5_)Sm(THF)}_2_(µ-η^2^:η^2^-N_2_Ph_2_)_2_], (**XIV**) [49]. Figure generated from CCDC obtained coordinates. Atoms of arbitrary size. H atoms are omitted for clarity.

Bi-metallic reduced azobenzene complexes have exhibited more strongly reduced azobenzene fragments, with N=N bond lengths ranging between 1.44(1) Å to 1.48_9_ Å as observed in [{(C_5_Me_5_)Sm(THF)}_2_{µ-η^2^:η^2^-N_2_Ph_2_}_2_], (**XIV**), and [{(C_5_H_5_)(THF)Lu}_2_{µ-η^2^: η^2^-N_2_Ph_2_}_2_], (**XXV**) [54], respectively, except for [{(C_5_Me_5_)Sm}_2_(μ-η^1^:η^1^-N_2_Ph_2_)], (**X**) discussed above, which did not exhibit N=N lengthening (1.25(1) Å). In most cases, the reduced azobenzene ligand bridges the lanthanide atoms in a µ-η^2^:η^2^ bonding motif, such as in [{(C_5_Me_5_)Sm(THF)}_2_(µ-η^2^:η^2^-N_2_Ph_2_)_2_], (**XIV**), shown in Figure 12.

**Figure 13:**
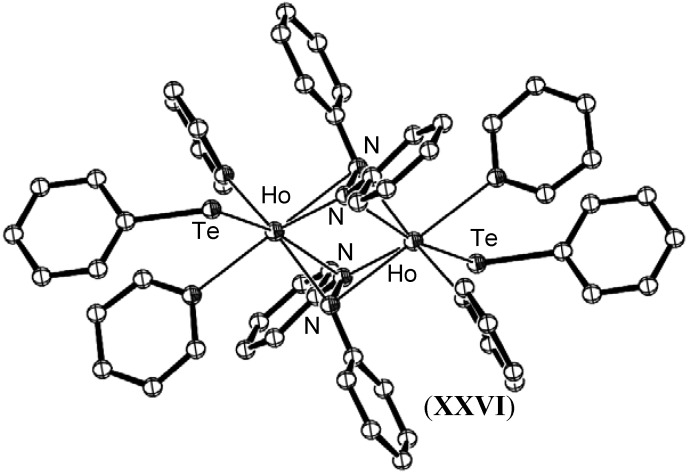
Molecular structure of [{Ho(PhNNPh)(TePh)(C_6_H_5_N)}_2_], (**XXVI**) [55]. Figure generated from CCDC obtained coordinates. Atoms of arbitrary size. H atoms, two pyridine molecules of crystallisation and one of the two independent molecules in the asymmetric unit are omitted for clarity.

Lanthanide(0) metal reagents have been used in the reduction of mixtures of PhNNPh/PhEEPh with the aim of cleaving the azo bond to give imido species. However bimetallic µ^2^- bridged azo compounds resulted, with cleavage of the chalcogen bond observed [55]. Complexes of this nature, such as [{Ho(PhNNPh)(TePh)(C_6_H_5_N)}_2_], (**XXVI**), shown in Figure 13 contained two dianionic reduced azo fragments distinct from the monoanionic reduction of azobenzene in [{(C_5_Me_5_)Sm(THF)}_2_(µ-η^2^:η^2^-N_2_Ph_2_)_2_], (**XIV**).

Azobenzene reductions utilising Ln(III) based reagents have also been reported recently. The reduced dinitrogen complexes such as [{(C_5_Me_5_)_2_(THF)La}_2_(µ-η^2^:η^2^-N_2_)], (**XXVII**), facilitated the reduction of azobenzene to give the µ-η^2^:η^2^ complex, [{(C_5_Me_5_)La(µ-η^2^:η^2^-PhNNPh)(THF)}_2_], (**XXVIII**). In this instance, the lanthanide(III) dinitrogen complex acts as a lanthanide(II) synthon, *via* the liberation of the N_2_^2-^ moiety as N_2_ itself [43].

## 6. Reactions of R(H)N-N(H)R Species

**Scheme 5:**
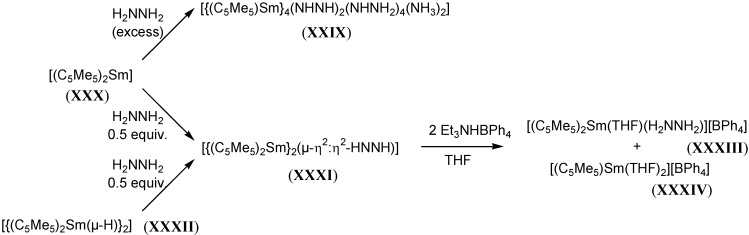
Reactivity of hydrazine with samarium pentamethylcyclopentadienyl complexes.

Relative to the reductions of N≡N and RN=NR species as described above, reactions of organolanthanide reagents with N-N moieties are limited. An unsubstituted hydrazido complex, [{(C_5_Me_5_)Sm}_4_(NHNH)_2_(NHNH_2_)_4_(NH_3_)_2_], (**XXIX**), synthesised from the reaction of [(C_5_Me_5_)_2_Sm], (**XXX**) with excess hydrazine has been reported [56], as has the generation of a dimetallated Sm hydrazine complex, [{(C_5_Me_5_)_2_Sm}_2_(µ-η^2^:η^2^-HNNH)], (**XXXI**) from the reaction of [{(C_5_Me_5_)_2_Sm(µ-H)}_2_], (**XXXII**) with hydrazine [35]. Protonation of hydrazine complex **XXXI** led to the isolation of the neutral hydrazine adduct, [(C_5_Me_5_)_2_Sm(THF)(H_2_NNH_2_)][BPh_4_], (**XXXIII**) [57]. The reactivity of hydrazine with the pentamethylcyclopentadienyl samarium systems is summarised in Scheme 5.

**Figure 14:**
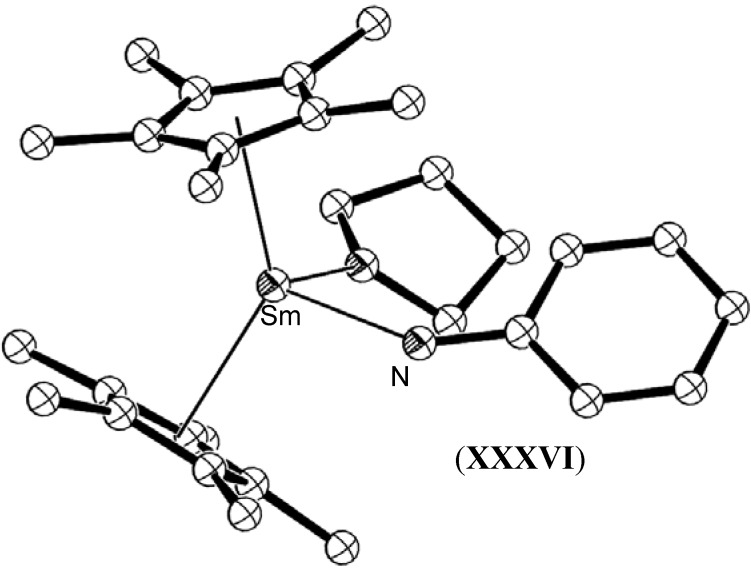
Molecular structure of [(C_5_Me_5_)_2_Sm(N(H)Ph)(THF)], (**XXXVI**) [35]. Figure generated from CCDC obtained coordinates. Atoms of arbitrary size. H atoms are omitted for clarity.

**Figure 15:**
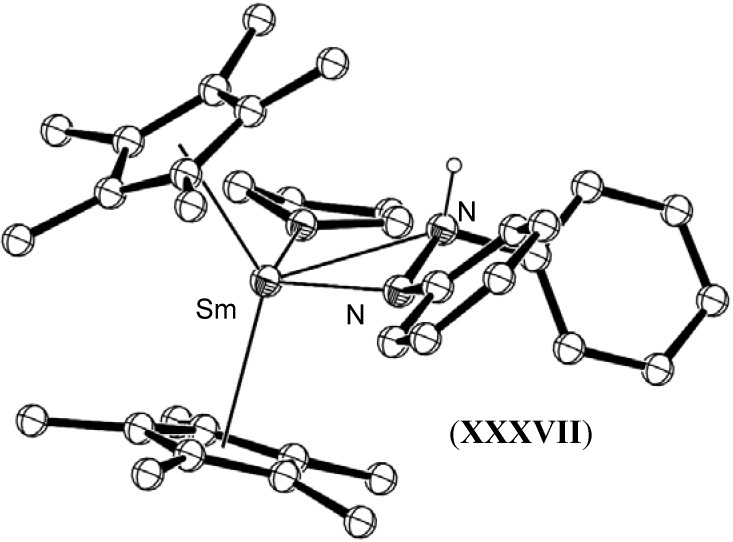
Molecular structure of [(C_5_Me_5_)_2_Sm{η^2^-PhNHNPh}(THF)], (**XXXVII**) [35]. Figure generated from CCDC obtained coordinates. Atoms of arbitrary size. Non-hydrazine H atoms are omitted for clarity.

Evans has also reported the reduction of diphenylhydrazine by [(C_5_Me_5_)_2_Sm(THF)_2_], (**XXXV**) and [(C_5_Me_5_)_2_Sm], (**XXX**) to yield the cleaved species [(C_5_Me_5_)_2_Sm(N(H)Ph)(THF)], (**XXXVI**), and the non-cleaved THF adduct of the metallated species [(C_5_Me_5_)_2_Sm{η^2^-PhNHNPh}(THF)], (**XXXVII**), shown in Figure 14 and Figure 15, respectively [35]. The isolation of **XXXVI** demonstrated the important capability of lanthanide reagents to facilitate the final reduction of the dinitrogen moiety to give mono-nitrogen complexes as required by the Chatt cycle.

## 7. Mono-Nitrogen Containing Lanthanide Complexes

**Figure 16:**
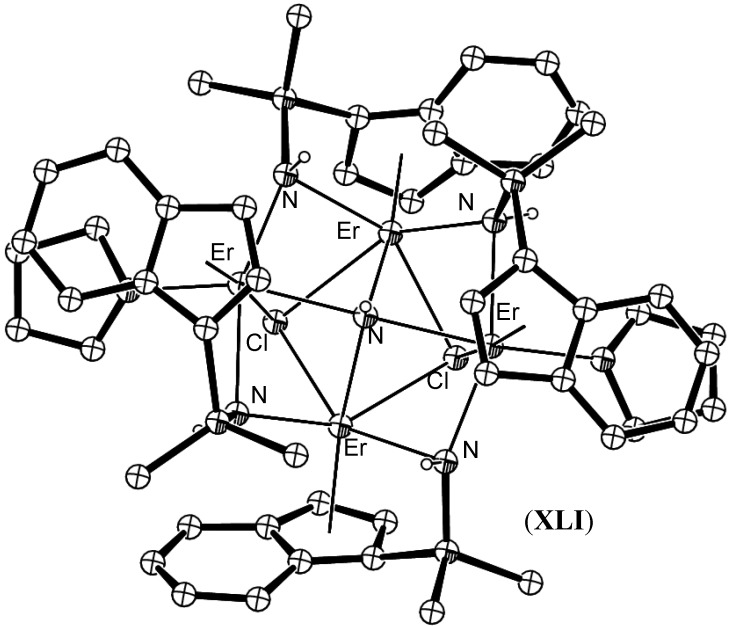
Erbium imide cluster **XLI** [61]. Figure generated from CCDC obtained coordinates. Atoms of arbitrary size. Non-nitrogen H atoms are omitted for clarity.

Lanthanide amine and amide compounds have been reported in abundance. However, imide species, which are amide precursors in the Schrock and Chatt cycles, have been reported sparingly in the literature [58,59,60,61,62,63,64,65,66,67]. Attempts to isolate a terminal lanthanide imide complex have as yet been unsuccessful. Schumann *et al.* reported the isolation of tetranuclear Sm(III), **XXXVIII** and Yb(III), (**XXXIX**) complexes containing an imide ligand from the reduction of azobenzene by the naphthalide complex (C_10_H_8_)Ln(THF)_n_ [58,59]. Xie *et al.* have reported a series of µ_4_-imido clusters of Gd, (**XL**), Er, (**XLI**) and Dy, (**XLII**) from the reaction of Me_2_Si(C_9_H_7_)(C_2_B_10_H_11_) with 4 equivalents of NaNH_2_, followed by treatment with one equiv of LnCl_3_ [60,61,62,63], Figure 16. µ_3_-Imido containing neodymium complexes of NPh with varying nuclearity have been reported by Ephritikhine using the magnesium transfer reagent [{(PhN)Mg(THF)}_6_] yielding THF and pyridine adducts with Nd_4_(NPh)_4_I_4_ and Nd_6_(NPh)_8_I_2_ cages [67,68]. This transfer reagent proved unsuccessful in our hands in attempts to access imido species using a sterically strained macrocycle as a supporting ligand [69,70].

**Figure 17:**
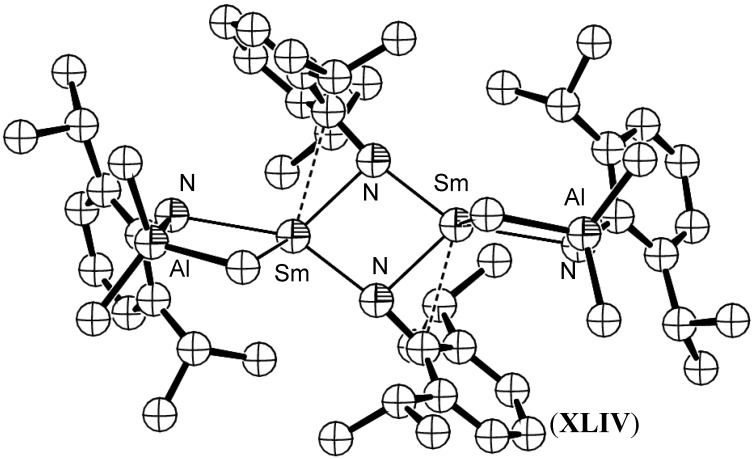
AlMe_3_ retained, nitrogen bridged samarium imide complex **XLIV**. Figure generated from CCDC obtained coordinates. Atoms of arbitrary size. H atoms omitted for clarity [65].

**Figure 18:**
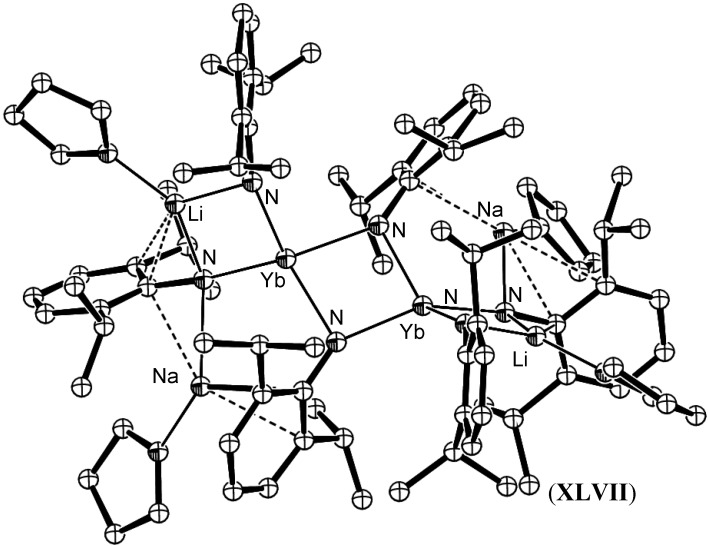
Ytterbium imide complex **XLVII** resulting from deprotonation by *n*-BuLi [66]. Figure generated from CCDC obtained coordinates. Atoms of arbitrary size. H atoms are excluded for clarity. Amido H atoms were inferred but were not located.

Hou *et al.* have reported the isolation of (^-^NC(H)Ph) complexes *via* the addition of Ln-H units across the C≡N bond in benzonitrile. The resulting methylene imide fragment formed a Ln_4_N_4_ cubane core [62,63]. A similar Ln_4_N_4_ cubane core was observed recently in the unique reductive cleavage of azobenzene by the bis(amidinate) Sm complex [Me_2_Si{NC(Ph)N(2,6-*i*-Pr_2_Ph)}SmI_2_Li_2_(THF)(Et_2_O)_2_] (**XLIII**). The resulting imido-bridged lanthanide cubane cluster represents the first such complex to result from N=N cleavage [64]. Gordon *et al.* synthesised a bis(µ^2^-imido) complex [(µ-ArN)Smµ-NHAr)(µ-Me)AlMe_2_]_2_, (**XLIV**), (Ar = 2,6-(*i*-Pr)_2_C_6_H_3_) *via* the reaction of [Sm(µ-NHAr)(NHAr)_2_]_2_, (**XLV**), with AlMe_3_ [65], Figure 17. In this instance, two equivalents of AlMe_3_ deprotonated the precursor amide moiety and a further two equivalents were incorporated into the dimeric complex to sterically shield the lanthanide centres. A similar approach has been used by Xie *et al*. to yield mixed amido-imido-ytterbium complexes *via* deprotonation of [(*i*-Pr_2_C_6_H_3_NH)_2_Yb(µ-N(H)-*i*-Pr_2_C_6_H_3_)_2_Na(THF)], (**XLVI**) with *n*-BuLi. A complex heterometallic Yb/Na/Li cluster, (**XLVII**) resulted [66], Figure 18.

In each of the reported imido syntheses to date, the final outcomes could not have been easily predicted. However, the isolation of these complexes has led to the prediction that organolanthanide complexes containing bulky non-participating ligands and mixed anilido-alkyl ligands should yield imido complexes in a directed manner [71].

## 8. Conclusions

The diverse chemistry reviewed in this article has clearly shown that the lanthanide metals are capable of stabilising a wide range of nitrogen- and dinitrogen-based ligands related to the conversion of nitrogen into ammonia. The narrow range of oxidation states available to the lanthanide metals has resulted in multi-nuclear and mixed-metals complexes dominating the range of (N_2_)^n-^ species that have been discovered, some of which are unknown to transition metal chemistry. Mononuclear lanthanide species feature more often in complexes featuring derivatives that have been postulated and/or isolated later in the Chatt and/or Schrock cycles based on transition metal chemistry. It is acknowledged that little has thus far been achieved in regard to driving the various complexes around the Chatt- or Schrock-type catalytic cycles and that most of the complexes presented herein represent alkyl/aryl substituted analogues of these species. These last raised issues are major challenges to be addressed in the future for this field in relation to any potential development of metal-free dinitrogen derivatives based on lanthanide metal mediated processes. Nevertheless, the outcomes presented so far will no doubt continue to be built on and a wealth of interesting and structurally diverse new chemistry will be established.
